# Testicular biohistochemical alterations following experimental varicocele in rats 

**Published:** 2012-05

**Authors:** Mazdak Razi, Rajab Ali Sadrkhanloo, Hassan Malekinejad, Farshid Sarrafzadeh-Rezaei

**Affiliations:** 1 *Department of Comparative Histology and Embryology, Faculty of Veterinary Medicine, Urmia University, Urmia, Iran.*; 2 *Department of Pharmacology and Toxicology, Faculty of Veterinary Medicine, Urmia University, Urmia, Iran.*; 3 *Department of Clinical Science, Surgery Division, Faculty of Veterinary Medicine, Urmia University, Urmia, Iran.*

**Keywords:** *Varicocele*, *Cytoplasmic carbohydrate*, *Lipid foci*, *Lipase*, *Alkaline-phosphates*, *Testis*

## Abstract

**Background**: The exact pathophysiology of testicular degeneration, following varicocele has not been completely understood yet.

**Objective:** The current study was designed to determine the effect of varicocele on germinal epithelium (GE) cytoplasmic biohistochmical alterations.

**Materials and Methods:** To follow-up this study, left varicocele was induced in test groups. Non-varicocelized rats were served as control-sham (n=6). Following 4, 6 and 8 months, right and left testes were dissected out and the blood serum sample was taken. The GE cytoplasmic carbohydrate, lipid accumulation, lipase and alkaline-phosphates (ALP) ratios were analyzed. Serum levels of LH, FSH and testosterone were measured.

**Results:** Observations demonstrated that in varicocele-induced rats, the spermatogenesis cell lineage exhibited lower number of cells with periodic acid shift positive cytoplasm, higher number of cells with lipid and ALP positive stained cytoplasm in comparison to control animals. Lipase enzyme decreased by the time in the test animals. In varicocelized groups the number of Leydig cells decreased in to 2.25±0.41 and 1.16±0.75 per one mm^2^ in left and right testicles respectively after 8 months, and these cells demonstrated an ALP positive feature. In test groups, the serum levels of LH and FSH reduced into 1.12±0.01 and 2.03±0.05 ng/ml respectively after 8 months. Although testosterone level diminished by the time in the test animals, and this decreasing was significant (p=0.031) after 8 months (3.08±0.10 ng/ml).

**Conclusion:** Our results suggest that following varicocele induction major alterations occur in GE, which may lead to loss of GE cells physiological function and ultimately result in fertility problems.

## Introduction

According to the epidemiological studies and hospital reports the clinical varicocele is observed in 10-20% of the general male population, in 35-40% of men with primary infertility and in up to 80% of men with secondary infertilities (1, 2). With recent advances in diagnostic techniques and widespread application of scrotal ultrasonography and color Doppler imaging, varicoceles are being reported in up to 91% of sub-fertile cases, most of who were previously regarded as having idiopathic etiology (3, 4). Semen quality uniformly ebbs in animals and humans with varicoceles (5). In order to declaring this impairment a number of theories have been suggested and the pathophysiology of varicoceles explained. 

Recently, in spite of various reports, the pathogenetics mechanisms by which varicocele induce testicular degeneration, spermatogenesis arrest and finally infertility are not completely understood. The suggested mechanisms include; reflux of toxic metabolites from adrenal and/or renal origin, impairment of the hypothalamic-gonadal axis, venous stasis leading to testicular hypoxia and elevation of temperature in testicles (6, 7). 

However, the cytoplasmic biochemical alterations in germinal epithelium (GE) and the role of inflammation in spermatogenesis and spermaiogenesis processes are enigmatic. It is well known, the cytoplasmic carbohydrates (mainly glucose) are the source and preliminary origin to supply required energy to the most of biochemical activities. Any disruption in carbohydrates metabolism and/or transport through GE can influence their mitotic and biological activities, which in turn can lead to spermatogenesis arrest in seminiferous tubules (4). The inhibition of testosterone synthesis in rats with surgically-induced varicoceles was shown to be essential to decrease the function and activity of the enzyme 17, 20 desmolase (8). 

In corroborating of this report, the previous studies indicated that the intra-testicular testosterone decreases in varicocele patients (9). There are different theories which are illustrating that the disruption between hypothalamus- gonadal axis following varicocele is able to affect the testosterone down-surging (6, 7). 

The notable question is that, if the negative feedback of Leydig cells for gonadothrophic hormones in early stages of varicocele is the main reason for endocrine failure or the varicocele affects the testicular endocrine system in primary stages and by the time the hypothalamus-gonadal axis enhances the impairments. On the other hand the histological examination of the Leydig cells for cytoplasmic ALP (as a well-known marker for inflammation) and also for lipid foci (as a marker for cellular steroidogenesis) can be helpful in order to have a better understanding of hormonal alterations. 

Thus the primary aim of the present study was to use especial techniques to illustrate the biohistochemical alterations of cytoplasmic carbohydrate supplement, unsaturated fatty acids (lipid foci as an alternative source of energy for carbohydrates), and cytoplasmic lipase enzyme (necessary intracellular enzyme for lipids metabolism). Additionally tissue alkaline-phosphates (ALP) as a biomarker for inflammation in seminiferous tubules (STs) were performed.

The second aim of the present study was to evaluate the serum level of testosterone, LH and FSH in experimentally-induced varicocele animals and to investigate the relationship of testosterone, LH and FSH deprivation with histopathological changes in testes. We also sought to analyze probable pathological changes in Leydig cells numbers in one mm^2^ of the interstitial connective tissue of both right and left testes in order to identify and compare the severity of varicocele impacts on both side testicles.

## Materials and methods

**Animals**

Twenty four mature male Wistar rats, 10 weeks olds and weighting 200±14 gr were used in order to perform interventional-experimental study. The rats were purchased from the Animal Resources Center of Faculty of Veterinary Medicine, Urmia University, Iran and they were acclimatized in an environmentally controlled room (temperature: 20-23^o^C, and 12h light/12h dark). Food and water were given ad libitum. In this study all experiments which conducted on animals were in accordance with the guidance of ethical committee for research on laboratory animals of Urmia University.

**Varicocele induction**

Following a week acclimation, the animals were assigned into four groups (n=6) as control-sham and test groups. The test animals sub-grouped according to the month of termination of the study as 4 months varicocele (n=6), 6 months varicocele (n=6) and 8 months varicocele (n=6) groups. In test group left varicocele was induced as previously reported (10). 

In brief; to reduce the renal vein to an external diameter of 1 mm, left renal vein ligation was performed at a direct medial to the junction of the adrenal and spermatic veins. Then the anastomotic branch between the left testicular vein and the left common iliac vein was ligated. The control-sham group anesthesized and only underwent to a simple laparatomy and no vein ligation was performed on these animals.

**Histochemical study**

Both left and right testes were dissected out and underwent to a histochemical studies. The specimens were freshly cut with frozen section and in order to analyze the testicular germinal epithelium carbohydrate ratio, periodic acid shiff (PAS) special staining technique was conducted on specimens. Furthermore the Sudan-Black B (SB) staining was performed to evaluate the rate of lipid foci supplement in GE of the both test and control-sham animals and to identify the Leydig cells cytoplasmic bio-steroid supplement. 

The alkaline-phosphates staining (ALP) was conducted to demonstrate the ratio of this enzyme as a biomarker for inflammation. The lipase enzyme staining was performed to evaluate any alterations in germinal cells cytoplasmic lipase. The number of Leydig cells per one mm^2^ of interstitial connective tissue was evaluated by 100 square lens devise (Olympus, Germany). All of the specimens were studied by multiple magnifications (400X and 1000X). 

In order to obtain statistically comparable data from histochemical examinations, the seminiferous tubules were classified in two types; type I: the tubules in which all of the germinal cells were staining positive, type II: the tubules in which half of the cells stained positive (9). 

**Serum sampling and hormonal assays**

Blood samples from corresponding animals were collected and serum samples were prepared with centrifugation (3000 g for 5 min), and subjected to assessment of the serum level of LH, FSH and testosterone. To measure the FSH and LH levels, radioimmunoassay (RIA) method was conducted according to the manufacture's structures by using the kits of WHO/Sigma Asso-RFGC-78/549 and WHO/Sigma Asso-RLGC-80/552, respectively. 

Testosterone was assessed by using immunoradiometric methods, using the kits of WHO/Sigma Asso-RTGC-768/98. The intra-assay coefficients variance (for 10 times) for FSH, LH and testosterone were; 3.56, 2.64, and 5.9 respectively and inter-assay coefficients variances of 8.98 (for 10 times), 7.52 (for 10 times) and 6.23 (for 10 times), were found for FSH, LH and testosterone, respectively.

**Statistical analysis**

All results are presented as mean±SD. Differences between quantitative histochemical, biochemical and morphometric data from the control-sham group and test groups were analyzed with two-way ANOVA, followed by Bonferroni test, using Graph Pad Prism, 4.00, p<0.05 was considered as significant differences.

## Results

**Effect of varicocele on cytoplasmic carbohydrate**

**Figure 1:**
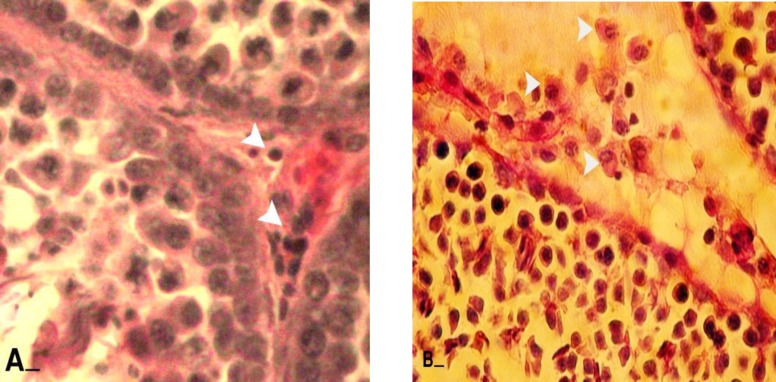
Cross sections from testes; (A) control group: note white head arrows indicating Leydig cells which are located close to the blood vessels. Leydig cells are presented faint reaction to PAS staining. (B) varicocele-induced group: note the head arrows showing Leydig cells which are separated in edemic interstitial connective tissue. Most of these cells are presenting dense PAS reaction. PAS staining, (600X), scale bar: 0.2mm

Light microscopic analyses showed that in varicocele-induced rats the spermatogonia and spermatocytes were revealed with low cytoplasmic carbohydrate ratio. This impairment was progressed by the time. Observations demonstrated that in varicocele-induced rats the majority of Leydig cells were manifested with dense PAS stained cytoplasm and rarely these cells showed faint PAS reaction. In contrast in control animals these cells were illustrated with faint cytoplasmic carbohydrate ratio and in contrary to the test groups, Leydig cells were located closely to the blood vessels and they were found in 6.85±1.21 cells per one mm^2 ^(Figure 1). 

**Figure 2:**
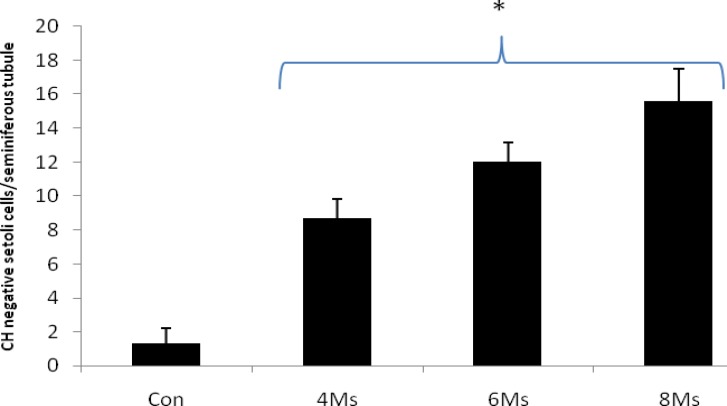
Mean average of PAS negative Sertoli cells per one seminiferous tubules in different test and control-sham groups. Star is indicating significant differences (p<0.032; 6 months vs. 4 months, p<0.041; 8 months vs. 6 months, p<0.037; 8 months vs. 4 months, p<0.001; 4 months vs. control, p<0.001; 6 months vs. control, p<0.001; 8 months vs. control) between all test groups with each other and with control-sham. All data are presented in Mean±SD.

**Figure 3:**
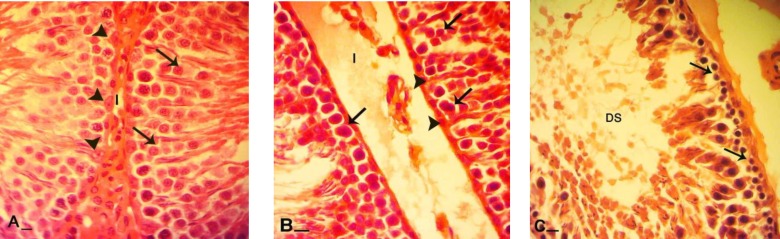
Cross sections from testes; (A) control group: note interstitial connective tissue (I) without edema, Sertoli cells with dense stained cytoplasm (head arrows), spermatogenesis cell lineage with powerful reaction for PAS staining which are indicating high cytoplasmic carbohydrate supplement. Comparing right testis (B) of varicocele-induced rats with left (C) indicates that cellular layers are significantly decreased, and spermatogenesis cell lineage in right testis (arrows) present stained cytoplasm while same cells (arrows) in left testis are remained unstained. Note to considerable edema in interstitial connective tissue (I) in figure B. In figure C seminiferous tubules depletion was occurred (DS). PAS staining, (A and B, 600X and C 400X), scale bar: 0.2mm

Analyses showed that in control group numerically lower Sertoli cells exhibited with faint PAS stained cytoplasm, while in test animals higher numbers of Sertoli cells were detected with faint cytoplasmic carbohydrate ratio (Figure 2). Comparing reaction density between control and varicocelized groups showed that in varicocele-induced animals the second three-cellular layers of GE were revealed with a faint PAS stained cytoplasms, which in contrast the control animals were manifested with low reaction sites in the same layers (Figure 3).

**Influences of varicocele on cytoplasmic lipid accumulation**

Histochemical observations demonstrated that in varicocele-induced animals the lipid accumulation was increased in spermatogenesis cell lineage. Accordingly the majority of spermatogonia cells were presented with dense lipid foci in their cytoplasm. By the time the lipid accumulation was increased and approximately most of the cells in different cellular layers of GE showed the SB-B stained cytoplasms. Right testes in varicocelized animals showed statistically significant lower percentage of type I tubules in comparison to the left testes (p=0.041). 

**Figure 4:**
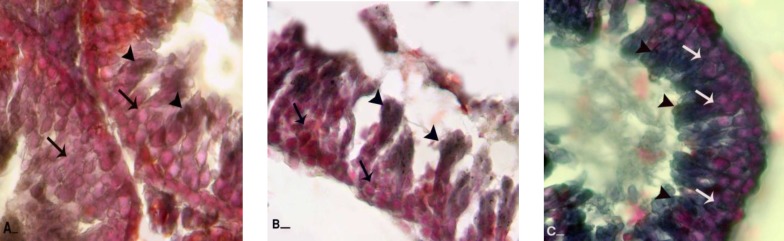
Frozen section from testes; (A) control group: note spermatogenesis cell lineage with negative Sudan-Black B stained cytoplasms (arrows) and spermaiogenesis area is appeared with dense reaction sites (head arrows). Comparing right testis from varicocele-induced rats (B) with left testis (C) and control group indicates that in right testis spermatogenesis cells lineage are presented with faint lipid stained cytoplasms (arrows) and spermayogenesis area (head arrows) stained densely, while left testis is manifested with darkly stained cells in all cell lineage (arrows and head arrows). Sudan Black B staining, (A and C, 400X and B, 600X), scale bar: 0.2mm.

In control group cytolplasmic lipid agglomeration was only detected in spermaiogenesis cell lineage. Statistically significant (p=0.047) low cells with SB-B positive cytoplasm were detected in spermatogenesis cells lineage of the control testes (Figure 4). The Leydig cells in varicocele- induced group were detected with very faint reaction sites for SB-B staining but in the control rats these cells demonstrated a clear stained cytoplasm. 

**The relationship between varicocele and cytoplasmic lipase modification**

**Figure 5:**
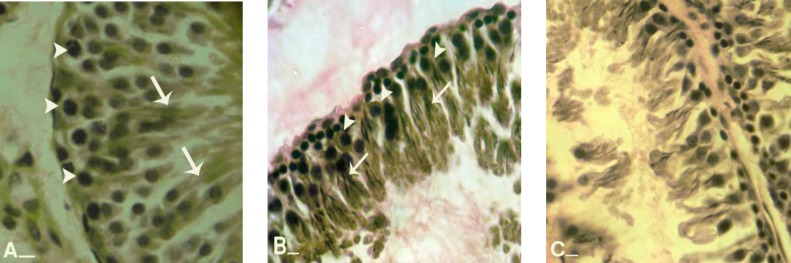
Frozen sections from testes; (A) control group: note head arrows which are indicating spermatogonia cells with negative reaction for lipase staining and spermaiogenesis area with dense lipase stained cytoplasms (arrows). (B) Right testis: note spermatocytes type I with dark lipase positive cytoplasms (head arrows) and cells in spermaiogenesis area with lipase positive sites (arrows). (C) Left testis after 8 months, note all cell types with very week stained cytoplasms. Lipase staining, (600X), scale bar: 0.2mm.

Cytoplasmic lipase normally was observed in spermaiogenesis cells lineage in the control group and this situation was constant in entire experiment period. Whereas the animals in test groups showed the high lipase stained sites in the cytoplasm of spermatogenesis cells lineage which was decreased by the time. Accordingly the left testes of the 8 months varicocele-induced animals exhibited very low response for lipase staining. In 4 and 6 months varicocele-induced rats the lipase reaction was seen in approximately all GE layers (Figure 5). Comparing lipase modification between left and right testes showed that the right testicles demonstrated the lower modification in comparison to left ones and the percentage of type I tubules decreased in 8 months varicocele animals in comparison to other test and control-sham groups.

**ALP alterations in varicocele**

**Figure 6:**
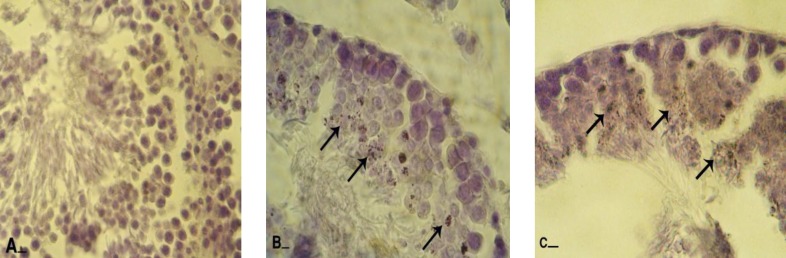
Frozen sections from testes; (A) control group: all germinative epithelium layers are presented with negative ALPO reaction. Comparing right testis (B) with left testis (C) illustrates that there is no considerable difference between the numbers of cells with ALPO stained cytoplasms. Most of the cells in upper layers are ALPO positive (arrows). Alkaline-phosphates staining, (400X), scale bar: 0.2mm.

**Figure 7:**
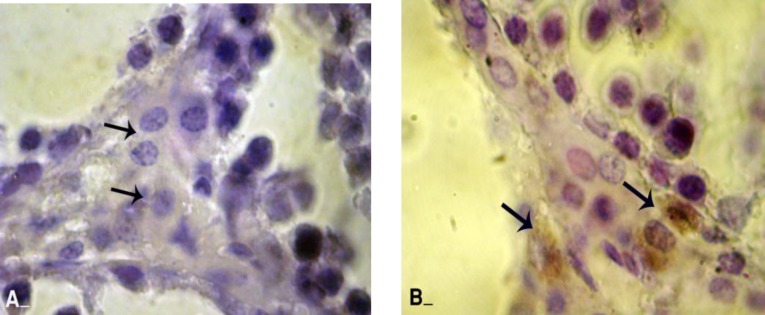
Frozen sections from testes; (A) control group: note the Leydig cells (arrows) with normal appearance with unstained ALPO. (B) Varicocele group: majority of Leydig cells are showed with dense ALPO sites (arrows). Alkaline-phosphates staining, (600X), scale bar: 0.2mm

Light microscopic analyses showed that in varicocele animals the ALP was significantly enhanced in majority of the STs. This impairment was mainly observed in disrupted epithelium. By the time the number of cells which were detected with ALP- positive reactions increased in the test animals. Although the right testes of varicocele-induced animals showed more cellular layers in comparison to the left, ALP reaction however was the same and most of the disrupted cells and especially those in upper layers revealed with ALP positive reacted sites. The testes from control animals showed statistically significant (p=0.045) lower percentage of tubules with type I features in comparison to all test groups (Figures 6 and 7). All data for histochemical studies in quantitative form are presented in table I.

**Varicocele influences the number of normal Leydig cells per testicle**

**Figure 8:**
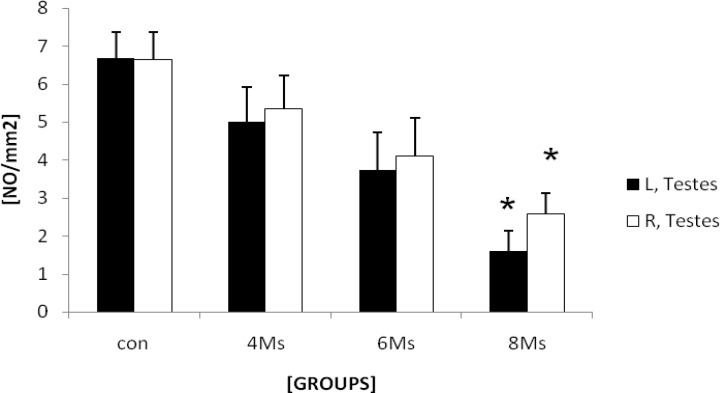
Mean average of Leydig cells number per one mm^2^ of the interstitial connective tissue of left and right testes in different control and test groups. There are significant differences (p≤0.041) between right and left testicles data after 8 months and as well between Leydig cells number after 6 (p<0.001) and 8 (p<0.001) months with control-sham animals. All data are presented Mean±SD.

Histological analyses manifested that the numbers of Leydig cells were decreased by the time in to 2.25±0.41 and 1.16±0.75 per one mm^2^ in left and right testicles after 8 months respectively. This impairment was enhanced more considerably in 8 months varicocele animals. Comparing right and left testes with each other showed that the left testes were undergone to higher degeneration of Leydig cells (Figure 8).

**The effect of varicocele on serum levels of testosterone, LH and FSH**

**Figure 9:**
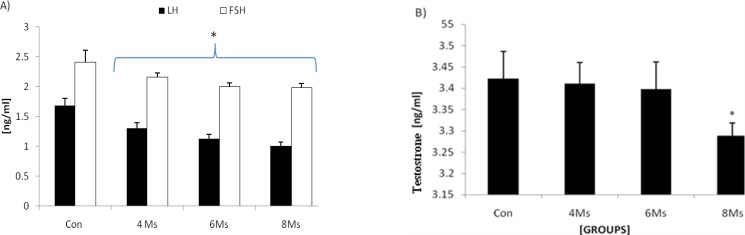
(A) Mean average of the LH and FSH serum levels and (B) mean average of testosterone serum level in different test and control groups. Stars are indicating significant differences, (p<0.045; 6 months vs. 4 months, p<0.031; 8 months vs. 6 months, p<0.043; 4 months vs. control, p<0.038; 6 months vs. control and p<0.037; 8 months vs. control) for FSH and (p<0.041; 6 months vs. 4 months, p<0.036; 8 months vs. 6 months, p<0.036; 4 months vs. control, p<0.031; 6 months vs. control and p<0.027; 8 months vs. control) for LH. There are significant differences (p<0.045; 8 months vs. 6 months, and p<0.031; 8 months vs. control) for testosterone. All data are presented in Mean±SD.

Hematological analyses revealed that the testosterone level was decreased but it was not statistically significant until 8 months. Rats in 8-moths varicocelized group showed a significant (p=0.031) decrease in testosterone level. The varicocelized rats demonstrated a sharp decline in the secretion of LH and FSH, accordingly the 8-months varicocele-induced rats were illustrated the lowest level of both hormones. The control animals showed higher serum level of testosterone, LH and FSH levels in comparison to test animals (Figure 9).

## Discussion

Varicocele is the main cause of primary and secondary male infertility. The high incidence of varicocele in men with secondary infertility and the fact that varicocele generally develops at the time of puberty, suggest that the presence of varicocele can cause a progressive decline in fertility. 

Although varicocelectomy is considered as a highly effective treatment of infertility, nevertheless due to varicocele a significant number of men remain infertile after varicocelectomy (10). There are reports indicating that patients with severely degenerated testicular cells following long time varicocele are not good candidates for varicocelectomy (6, 10, 11). 

Thus there is an increasing interest to develop methods to identify the exact patophysiology of varicocele in order to find the conservative models of therapy. In present study we aimed to show how experimentally-induced varicocele could exert pathological impact on the testes tissue in detail. To uncover the feature of varicocele-induced testicular damages various well established histopathological approaches were performed. 

Very early and remarkable finding of first step was that, varicocele induces degeneration in STs. We used the special method of the SB-B to evaluate the lipid accumulation in the cytoplasm of the GE of the STs and interstitial connective tissue of the testes. Comparing the reaction density for SB-B between the control and varicocelized animals indicated that varicocele-induced rats showed significantly increased lipid supplements in the GE of the STs especially spermatogenesis cell lineage. 

To explain how varicocele causes enhancement of lipid accumulation in mentioned cells, one should note that the lipid supplement in Sertoli cells differs depending on various conditions. For instance, when these cells phagocyte residual bodies or damaged cells, the ratio of lipids increases in the cytoplasm of them (12, 13). 

Our ALP staining was in good accordance with this hypothesis, because the varicocelized testes manifested with a powerful ALP reaction especially in disrupted cells. According to these findings it would be more logic to say that the number of inflammated (ALP positive cells) and disrupted GE cells increased in STs and eventually phagocytosis elevated. Whereas the first three layers of GE (the source cells for spermatogenesis) in the control rats were detected with PAS positive cytoplasms. We can hypothesis that carbohydrates (mainly glucose) are the main energy sources in these cells.

Previous studies showed that the glucose transporters are the main transferring way of glucose to the STs (10, 14). Thus any degeneration event could result in interruption of glucose passing-on to the STs and ultimately to GE. Thus this situation can suggests another hypothesis that following varicocele induction glucose transporting and/or metabolism is decreased in STs and pathophysiologically GE cells switched their energy source from glucose to lipids. Therefore cytoplasmic lipid foci increase in the cytoplasm of the cells especially those in first layers could be because of inadequate energy. At the same time, due to insufficient energy, these cells are not able to synthesize essential proteins, thus undergo to apoptosis and disruption in the STs of test animals (15-18). 

Histopathological studies in animals have shown that the varicocele induction inhibits spermatogenesis and degenerate the seminiferous tubules (5, 6, 10-21). Maintaining the serum level of LH and FSH at normal concentration is very important for initiating and supporting spermatogenesis. Hence degeneration of Sertoli cells and GE cells may be due to alteration in concentrations of circulating LH and FSH (22, 23). 

Our findings were in good accordance with these reports that SB-B staining in varicocele-induced rats showed high cytoplasmic lipid accumulation in Sertoli cells with faint lipase activity both in Sertoli, in disrupted and/or three first layers of GE cells. In contrast the Sertoli cells and spermatogenesis source cells in control group manifested the sharp PAS positive cytoplasm which indicates normal property. On the other hand biochemical analyses showed that in varicocele-induced rats the serum level of LH and FSH was significantly decreased. Varicocele causes GE disruption (24), and finally leads to tubular atrophy (9, 10, 25). 

The spermatogenesis and cellular integrity in mammals depends largely on testosterone production by Leydig cells in response to stimulation by FSH and LH. FSH increases Sertloi cell synthesis of an androgen binding protein needed to maintain the high concentrations of testosterone (22, 23, 26). According to previous reports the serum level of testosterone was reduced in men with long time varicocele and/or sexual insufficiency (17, 27, 28). Our SB-B staining corroborated and illustrated all these findings by showing Leydig cells with faint cytoplasmic steroid supplement and also the serum level of the testosterone decreased by the time in varicocele cases while the control animals showed inverse situation. 

At the same time, our histological analyses showed that in varicocele-induced groups the number of Leydig cells decreased by the time and these cells were revealed hypertrophic and accumulated locally. ALP staining confirmed our previous results and illustrated that the ratio of ALP in Leydig cells was increased remarkably in test groups. Thus, taken together we can conclude that following sever degeneration in Leydig cells (because of decreased LH and FSH), the intratesticular and serum testosterone reduction was happened and Sertoli cells underwent to a severe deterioration which in turn affected GE’s integrity in varicocele-induced rats.

## Conclusion

Following varicocele-induction the cells in spermatocytogenesis and spermatogenesis lineage in STs and Leydig cells in interstitial connective tissue possibly switch their energy source from glucose to lipids. Thus the inadequate energy supplement in a time-dependent manner results in lose of biological activities in order to use lipids as a alternative source of energy and ultimately leads to cellular degeneration. Moreover Leydig cells impairment in testosterone synthesis ruins the pathological process by affecting of the Sertoli cells.
